# Challenges in Predicting Estimated Glomerular Filtration Rate Slope Among Adult Patients With IgA Nephropathy: A Population-Based Cohort Study

**DOI:** 10.1177/20543581261468724

**Published:** 2026-09-30

**Authors:** Bryce Barr, Mark Canney, Navdeep Tangri, Lisa M. Lix

**Affiliations:** 1 Section of Nephrology, Department of Medicine, 12359 Max Rady College of Medicine, University of Manitoba, Winnipeg, MB, Canada; 2 Chronic Disease Innovation Centre, 60426 Seven Oaks General Hospital, Winnipeg, MB, Canada; 3 Department of Medicine, University of Ottawa, Ottawa, ON, Canada; 4 Ottawa Hospital Research Institute, Ottawa, ON, Canada; 5 College of Community and Global Health, Rady Faculty of Health Sciences, 8664 University of Manitoba, Winnipeg, MB, Canada; 6 George and Fay Yee Centre for Healthcare Innovation, 8664 University of Manitoba, Winnipeg, MB, Canada

**Keywords:** IgA nephropathy, prediction, epidemiology, eGFR slope, glomerulonephritis

## Abstract

**Background:**

Traditional epidemiologic studies and clinical trials in IgA nephropathy (IgAN) have focused on kidney failure or large declines in estimated glomerular filtration rate (eGFR) as primary outcomes. Two-year eGFR slope has emerged as a validated surrogate endpoint and a means of earlier risk stratification, yet no tools exist to predict eGFR slope in patients with IgAN.

**Objective:**

To develop and internally validate a prediction model for 2-year eGFR slope using routinely available clinical and pathological data in adults with IgAN.

**Design:**

Adults with biopsy-proven IgAN (2002–2021) were identified from the Manitoba Glomerular Diseases Registry and linked to population-based laboratory and administrative health data. Mixed-effects linear regression models containing demographic, clinical, and comorbidity covariates with random intercepts and slopes predicted eGFR slope. Four pre-specified models of increasing complexity were tested. Model performance was assessed using marginal and conditional R^2^, root mean squared error, calibration, and Bland–Altman analyses. Internal validation was performed using 10-fold cross-validation.

**Results:**

The cohort included 181 patients (median age 41 years [interquartile range (IQR) 31–54]); 137 (75.7%) cases with complete data were used in the primary analysis. Patients were high risk, with median eGFR 58 mL/min/1.73 m^2^ (IQR 35–86) and proteinuria 1.73 g/day (IQR 1.06–2.95) at biopsy. Higher index proteinuria was independently associated with faster eGFR decline (–1.47 mL/min/1.73 m^2^/year per 1 g/day, SE 0.66; p=0.009). A parsimonious model including time, age, sex, index eGFR, index proteinuria, and two-way interactions of time with eGFR and proteinuria had excellent fit (conditional R^2^ 0.95). Conditional (i.e., patient-specific) slope estimates, which incorporated patient-level random effects estimated from longitudinal eGFR measurements, closely approximated observed trajectories, with 88% of predicted slopes within 1 mL/min/1.73 m^2^/year of observed values. In contrast, marginal (i.e., population-average) predictions were poorly calibrated (11% within 1 mL/min/1.73 m^2^/year; calibration slope 1.58, 95% CI 0.71–2.44). Cross-validation demonstrated marked deterioration in conditional performance, while marginal predictions were stable but inaccurate.

**Limitations:**

Model performance was strongest when incorporating follow-up eGFR data obtained after the index date, which limits applicability for prediction in newly diagnosed patients. Sample size was constrained by missing data, and Oxford M and E scores were not available for all patients.

**Conclusions:**

While mixed-effects linear regression models accurately characterized observed eGFR trajectories, predictive performance relied on patient-specific random effects and may not generalize to new patients. Baseline clinical and pathologic variables could not accurately predict eGFR slope. These findings highlight both the promise and challenges of slope-based prediction in IgAN and underscore the need for larger cohorts with multiple eGFR measurements, and models that leverage prior patient data to improve out-of-sample performance.

## Introduction

Immunoglobulin A nephropathy (IgAN) is the most common primary glomerulonephritis worldwide and leads to chronic kidney disease (CKD) and kidney failure in a substantial proportion of affected individuals.^1-3^ The global incidence of IgA nephropathy is estimated at 2.5 cases per 100,000 persons per year. Within Canada, data from Manitoba show that 45% of affected patients progressed to kidney failure over a median follow-up of six years.^4,5^ Until recently, treatment options for IgAN were limited and supported by low-quality clinical trial data, with kidney failure (defined as estimated glomerular filtration rate (eGFR) <15 mL/min/1.73 m^2^ or receipt of kidney replacement therapy) serving as the primary outcome.^6-8^

Although many patients have reduced eGFR at diagnosis, progression to kidney failure often occurs over years or decades. Currently available prediction models in IgAN and CKD focus on outcomes such as a 50% decline in eGFR or kidney failure and may therefore underestimate risk among patients early in their disease course or with lower levels of proteinuria who are not expected to reach such outcomes over a 5-year prediction period.^9,10^ This gap in risk prediction is supported by recent epidemiologic studies extrapolating observed rates of eGFR decline, which suggest that most patients with IgAN will ultimately progress to kidney failure over their lifetime, even among groups traditionally considered low risk.^11,12^

In parallel, the landscape of IgAN therapeutic clinical trials has evolved. Regulatory acceptance of validated surrogate endpoints including short-term proteinuria reduction and 2-year eGFR slope by the United States Food and Drug Administration has enabled trials with shorter, predefined timelines and has accelerated therapeutic development, leading to approval of multiple new therapies in recent years.^13-16^ As a result, eGFR slope has become the preferred outcome in contemporary IgAN trials.

A model to predict eGFR slope is important for identifying patients with high lifetime kidney failure risk, even if short-term (i.e 5-year) risk is low. In addition, given changes in clinical trial design, identifying patients at risk of rapid eGFR decline who may not experience a kidney failure endpoint within the timeframe of a trial has implications for selecting patients for trial enrolment.

Accordingly, the aim of this study was to develop and internally validate a prediction model for 2-year eGFR slope in a population-based adult cohort with biopsy-proven IgAN.

## Methods

### Study Design and Setting

We conducted a population-based, retrospective cohort study of adults with IgAN in Manitoba, Canada. Patients were identified from the Manitoba Glomerular Diseases Registry, which captures all native kidney biopsies performed as part of routine clinical care in the province since January 2002.^5^ Pathology data from the registry were linked to administrative health and laboratory databases housed in the Population Research Data Repository at the Manitoba Centre for Health Policy (MCHP) at the University of Manitoba using a unique scrambled personal health identification number.

### Study Population

Adults aged 18 years or older with biopsy-proven IgAN between January 1, 2002 and December 31, 2020 were eligible for inclusion. Patients were excluded if they had a diagnosis other than IgAN, a clinical diagnosis of IgA vasculitis, could not be linked to the provincial health registry, or reached kidney failure within one year of biopsy. Kidney failure was defined as receipt of maintenance dialysis or kidney transplantation, identified using physician billing codes (Supplementary Table 1). This case definition has previously been validated in Manitoba.^17^ To ensure stable estimation of 2-year eGFR slopes, patients with fewer than three years of follow-up from biopsy or fewer than three eGFR measurements during follow-up were excluded. The latter criterion was chosen because reliable estimation of individual eGFR slope requires at least three measurements. The date of cohort entry was the date of kidney biopsy. Therapies for IgAN may have acute effects on eGFR that differ from their long-term effects, potentially violating linearity assumptions in eGFR slope models. To address this, the index date was defined as the date of the eGFR measurement closest to 365 days after biopsy and within a 90-day window of the 365 days. Follow-up continued for two years after the index date or until kidney failure, death, loss of health coverage, migration out of province, or study end (December 31, 2023). The study timeline is presented in Figure 1.

**Figure 1.**
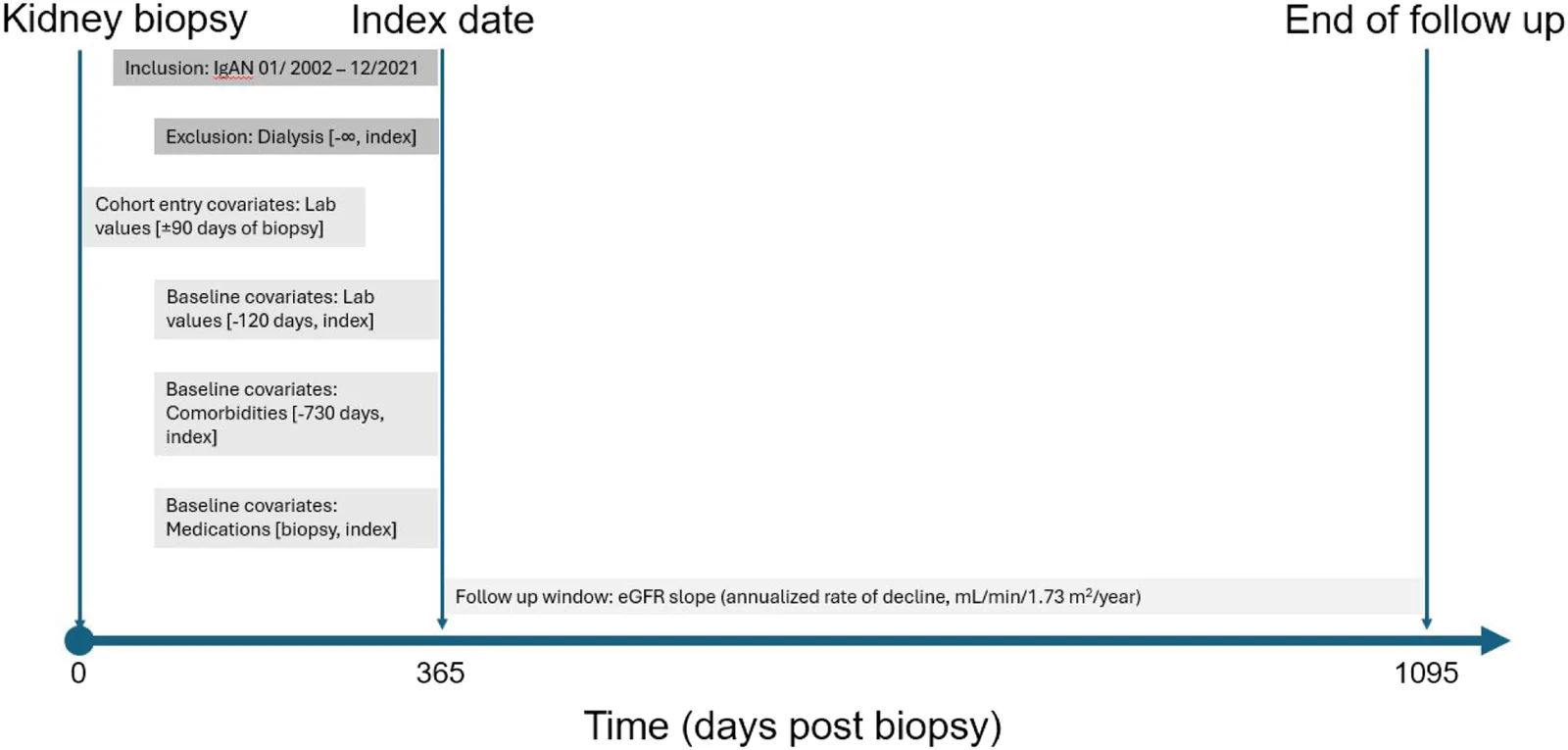
Overview of study design and covariate ascertainment

### Data Sources

Pathologic data were obtained from the Manitoba Glomerular Diseases Registry. Linked administrative data included laboratory results from Shared Health Diagnostic Services of Manitoba, socio-demographic and health insurance coverage (including loss of coverage due to death) from the Manitoba Health Insurance Registry, physician billing claims to capture comorbidities, and prescription drug data from the Drug Program Information Network to capture prescription of renin-angiotensin system inhibitors (RAASi) and glucocorticoids.

### Covariates

Baseline covariates were defined using data at or before the index date. Age and sex were obtained from provincial health insurance registry records. eGFR was calculated using the CKD-EPI 2021 equation.^18^ Cohort entry eGFR was defined as the measurement closest to the biopsy date within ±90 days. Index eGFR was defined as the measurement closest to, but not exceeding, 365 days post-biopsy, within 90 days. Proteinuria was expressed as 24-hour urine protein (g/day). When 24-hour collections were unavailable, spot urine protein-to-creatinine ratio (PCR) or albumin-to-creatinine ratio (ACR) values were converted to 24-hour equivalents using previously validated methods.^19-21^ Cohort entry and index proteinuria were defined using the same temporal windows as eGFR. Change in proteinuria and eGFR from biopsy to index date were evaluated as absolute differences. Hypertension and diabetes were defined using validated Canadian Chronic Disease Surveillance System algorithms based on the International Classification of Diseases, Ninth Revision, Clinical Modification (ICD-9-CM) and ICD-10-CA (Canadian version) codes (Supplementary Table 1).^22^ Use of RAASi was identified by prescriptions with Anatomical Therapeutic Chemical (ATC) codes beginning with C09, while glucocorticoid therapy was identified using ATC code H02.^23^ Treatment with either RAASi or glucocorticoids was defined as receipt of at least one prescription with a minimum duration of 30 days between the biopsy date and the index date. The Oxford classification was not routinely reported in our centre prior to 2016. However, the number of glomeruli including the number with segmental sclerosis and crescents are available in the registry data, as is the degree of interstitial fibrosis and tubular atrophy (IFTA). Accordingly, the Oxford S, T, and C lesions were reconstructed from raw pathology data using Oxford definitions.^24^ Due to low prevalence of C2 lesions, crescents were treated as a binary variable. Oxford M and E lesions were not available for patients prior to formal reporting of the Oxford classification and were therefore not included in the models.

### Outcome

The primary outcome was the 2-year eGFR slope, expressed in mL/min/1.73 m^2^ per year. The slope was the annualized linear rate of decline in eGFR from the index date to the last day of follow-up.

### Statistical Analysis

Study data were descriptively summarized using the median and interquartile range (IQR) for continuous variables and frequencies and percentages for categorical variables.

Mixed-effects linear regression models were fitted with eGFR as the dependent variable and time (in years) as the primary independent variable.^25^ All models included a random intercept and random slope for time to account for between-patient heterogeneity in baseline kidney function and rate of decline.

Candidate fixed-effect covariates included age, sex, index eGFR, index proteinuria, change in eGFR and proteinuria from biopsy to index, time from nephrology consultation to biopsy, glucocorticoid exposure, hypertension, diabetes, and available Oxford pathology features. Two-way interaction terms with time were tested for clinically relevant predictors, including index eGFR and index proteinuria.

Four pre-specified models of increasing complexity were evaluated: a parsimonious clinical model, a treatment and trajectory model, a full clinical model, and a clinicopathologic model (Supplementary Table 2). Model selection prioritized parsimony and predictive performance. Models were compared using Akaike (AIC) and Bayesian Information Criteria (BIC) to balance predictive performance and model parsimony.^26,27^ Model fit was assessed using marginal and conditional R^2^ and root mean squared error (RMSE).^28,29^ In mixed-effects models, the marginal R^2^ quantifies the proportion of variability in the observed data explained by the fixed effects, while the conditional R^2^ quantifies the proportion of variability explained by both fixed and random effects. The RMSE represents the average magnitude of prediction error between observed and predicted eGFR.

### Derivation and Interpretation of eGFR Slope Predictions

In these models, eGFR was modeled as a continuous longitudinal outcome, with time representing years since the index date. The estimated eGFR slope was calculated as the sum of the fixed effect of time, the fixed effect coefficients for all time-covariate interaction terms multiplied by that individual’s covariate values, and the patient’s random slope. We reported conditional (patient-specific) slopes, which included the random slope calculated using best linear unbiased predictors (BLUPs), and marginal (population-average) slopes, which set random effects to zero. Marginal slope predictions can be interpreted as the expected rate of eGFR change for a patient with given baseline characteristics, averaged across the population. Conditional slope predictions additionally reflect patient-specific deviations from the population-average trajectory, as estimated by the random effects. Because BLUP-estimated random effects were derived using all longitudinal eGFR measurements available during follow-up, conditional predictions should be interpreted as patient-specific fitted trajectories rather than prospective baseline predictions. The model equation and calculation of patient specific and marginal slopes are expressed mathematically in the Supplementary Appendix. Observed slopes were estimated separately for each patient using ordinary least squares regression of eGFR on time. Calibration plots were used to assess agreement between predicted and observed eGFR slopes across the range of model predictions,^30^ evaluating whether the model systematically over- or under-estimated kidney function decline at different levels of risk. Bland-Altman plots were used to examine individual-level agreement by visualizing the distribution of prediction errors and identifying overall bias and variability across the spectrum of eGFR slopes.^31^ We also assessed the proportion of predictions within 1 mL/min/1.73 m^2^/year of observed values.

### Internal Validation

Internal validation was performed using 10-fold cross-validation, with folds generated prior to model fitting. The dataset was randomly partitioned into 10 mutually exclusive folds of approximately equal size. Partitioning occurred at the patient level; all observations for a given patient were contained within a single fold. For each iteration, models were refitted using nine folds as the training set and evaluated in the remaining fold as the test set. Performance metrics were averaged across folds, and optimism was estimated by comparing apparent and cross-validated performance. Random effects for held-out patients were set to zero during cross-validation, resulting in population-average predictions.

### Missing Data

Missingness was present primarily in laboratory variables. Patterns of missing data were examined, and missingness was not consistent with the assumption of missing completely at random. Multiple imputation using chained equations was performed with five imputed datasets^32^; however, models fitted to imputed datasets demonstrated inferior performance and poorer calibration compared with complete case analyses. Differences in effect estimates between imputed and complete case models suggested sensitivity to imputation assumptions, likely reflecting informative missingness related to era and disease severity, making the assumptions underlying multiple imputation difficult to justify. Most importantly, missingness of index eGFR was prevalent. As index eGFR represents the first data point from which eGFR slope is derived, imputation of missing index eGFR values would effectively impute a component of the outcome itself, so even small errors in imputed values of the index eGFR may result in large distortions in eGFR slope. Complete cases were therefore used for the primary analysis.

The index date of 365 days post-biopsy was chosen to avoid non-linear effects of treatments prescribed soon after biopsy.^33^ As this date was chosen arbitrarily, a sensitivity analysis was performed whereby the models were re-fit with the index date set at 180 days post-biopsy, and the performance and fit of each model was compared to the primary analysis.

All analyses were conducted using R version 2025.05.1 (R Foundation for Statistical Computing, Vienna, Austria), including the lme4 and performance packages for mixed-effects modeling and model evaluation.

## Results

### Study Cohort

We identified 458 individuals diagnosed with IgAN between January 1, 2002 and December 31, 2020 in the Manitoba Glomerular Diseases Registry. Of those, 90 (19.7%) reached kidney failure within 1 year, 47 (10.3%) were followed for less than 3 years, and 140 (30.6%) had less than 3 eGFR measurements over the follow-up period, leaving 181 patients in the final cohort (Figure 2), with 137 (29.9%) complete cases. The median number of eGFR measurements within two years of the index date was 4 (interquartile range [IQR] 3–5) (Supplementary Figure 1).

**Figure 2.**
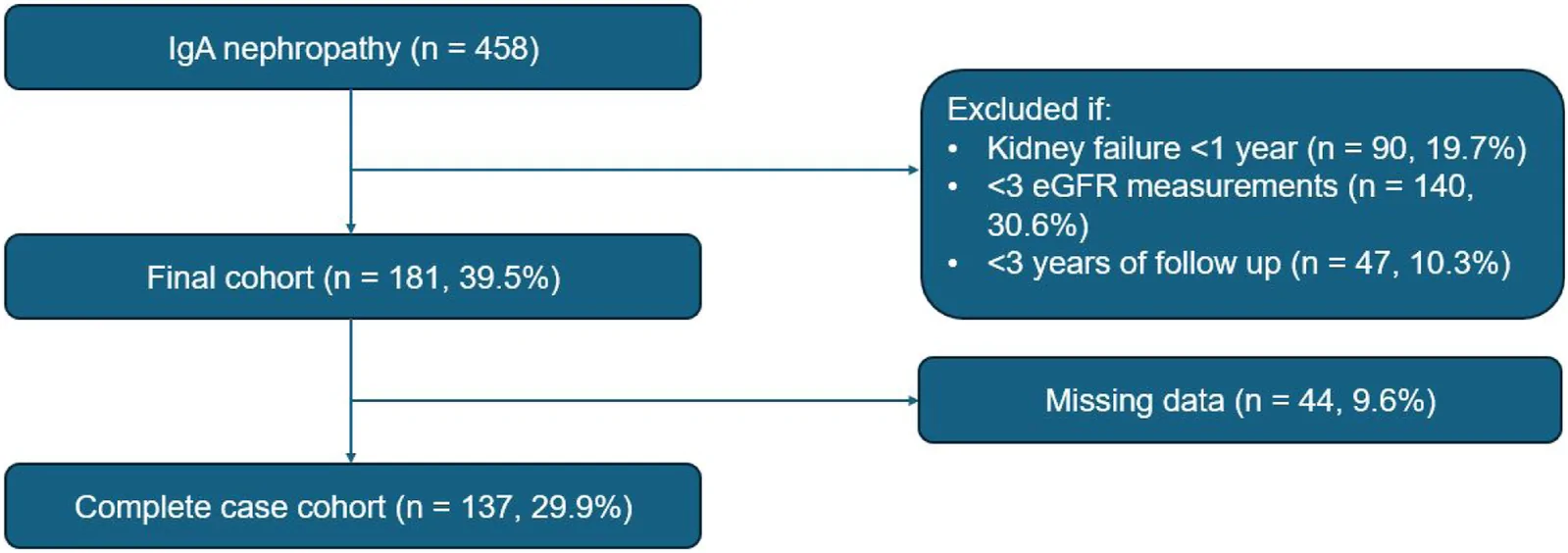
Cohort flow diagram

Baseline characteristics of the final cohort are summarized in Table 1. The median age was 41 years (IQR 31–54), and 56% of participants were male. Median eGFR at biopsy was 58 mL/min/1.73 m^2^ (IQR 35–86), and median proteinuria was 1.73 g/day (IQR 1.06–2.95). Compared to patients with three or more measurements, patients with two measurements were more frequently male, more often from Winnipeg (i.e., urban residents), with lower eGFR at both cohort entry and index dates. The characteristics of the final cohort were similar to those of complete cases (Supplementary Table 3).

**Table 1.** Baseline Characteristics of Patients With IgA Nephropathy by Study Era

Characteristic	Overall (n = 181)^1^	2002 to 2008 (n = 36)^1^	2009 to 2014 (n = 55)^1^	2015 to 2020 (n = 90)^1^
Age at cohort entry (years)^1^	41 (31, 54)	38 (26, 47)	44 (32, 58)	42 (31, 56)
Sex				
Male	101 (56%)	21 (58%)	31 (56%)	49 (54%)
Female	80 (44%)	15 (42%)	24 (44%)	41 (46%)
Location of Residence				
Winnipeg	108 (60%)	27 (75%)	33 (60%)	48 (53%)
Non-Winnipeg	73 (40%)	9 (25%)	22 (40%)	42 (47%)
SES Quintile				
1 (lowest)	62 (34%)	9 (25%)	19 (35%)	34 (38%)
2	36 (20%)	6 (17%)	13 (24%)	17 (19%)
3	30 (17%)	6 (17%)	7 (13%)	17 (19%)
4	36 (20%)	s	s	13 (14%)
5 (highest)	17 (9.4%)	s	s	9 (10%)
eGFR at cohort entry (mL/min/1.73 m^2^)^1^	58 (35, 86)	67 (30, 98)	52 (31, 80)	58 (39, 87)
Missing	13 (7.2%)	8 (22%)	s	s
Proteinuria at cohort entry (g/day)^1^	1.73 (1.06, 2.95)	1.71 (0.88, 2.85)	1.73 (1.04, 3.52)	1.77 (1.08, 2.61)
Missing	14 (7.7%)	9 (25%)	s	s
Index eGFR (mL/min/1.73 m^2^)^1^	51 (31, 81)	66 (40, 87)	43 (27, 71)	55 (33, 84)
Missing	26 (14%)	7 (19%)	s	15 (17%)
Index proteinuria (g/day)^1^	1.19 (0.53, 2.27)	0.70 (0.35, 2.22)	1.08 (0.47, 2.00)	1.61 (0.60, 2.56)
Missing	33 (18%)	8 (22%)	7 (13%)	18 (20%)
Nephrology consult to biopsy (days)	49 (8, 210)	38 (-16, 144)	62 (-7, 274)	49 (16, 233)
Diabetes	31 (17%)	s	10 (18%)	18 (20%)
Hypertension	83 (46%)	12 (33%)	25 (45%)	46 (51%)
RAASi treatment	160 (88%)	s	48 (87%)	78 (87%)
Glucocorticoid treatment	46 (25%)	12 (33%)	16 (29%)	18 (20%)
Oxford S				
0	50 (28%)	12 (33%)	16 (29%)	22 (24%)
1	131 (72%)	24 (67%)	39 (71%)	68 (76%)
Oxford T				
0	94 (52%)	25 (69%)	24 (44%)	45 (50%)
1	57 (31%)	11 (31%)	24 (44%)	22 (24%)
2	30 (17%)	0 (0%)	7 (13%)	23 (26%)
Oxford C (binary)				
0	132 (73%)	21 (58%)	39 (71%)	72 (80%)
1	49 (27%)	15 (42%)	16 (29%)	18 (20%)
Kidney failure status				
No kidney failure	148 (82%)	27 (75%)	46 (84%)	75 (83%)
Kidney failure	33 (18%)	9 (25%)	9 (16%)	15 (17%)
Vital status				
Alive	155 (86%)	28 (78%)	45 (82%)	82 (91%)
Dead	26 (14%)	8 (22%)	10 (18%)	8 (8.9%)
Follow-up time (years)^1^	8.2 (5.2, 12.6)	15.7 (14.5, 17.5)	10.7 (9.2, 12.8)	5.8 (4.2, 7.4)

SES, socioeconomic status; eGFR, estimated glomerular filtration rate; RAASi, renin-angiotensin-aldosterone system inhibitor; s, suppressed (cell size 5 or less).

^1^Median (Q1, Q3) are reported for continuous variables.

The number of patients diagnosed with IgAN increased over time, from 36 patients in 2002–2008 to 90 patients in 2015–2020. Patients diagnosed in earlier eras had lower median eGFR at biopsy and index and experienced higher rates of kidney failure and death during follow-up, consistent with longer observation time. Proteinuria at biopsy was similar across eras, but proteinuria at the index date was lowest in the earliest era (median 0.70 g/day, IQR 0.35-2.22) and highest in the most recent era (median 1.61 g/day, IQR 0.60-2.56). Use of renin–angiotensin system inhibitors was high and stable across eras (n = 160, 88%), whereas glucocorticoid use declined over time, from 12 patients (33%) in 2002-2008 to 18 patients (20%) in 2015-2020.

### Model Development and Evaluation

In addition to the fixed effect of time, a random intercept and random slope for time, the parsimonious model included fixed effects of index eGFR, index proteinuria, age, and sex, with two-way interactions for time with index eGFR and index proteinuria. Measures of model fit were similar across all candidate models, with marginal R^2^ values near 0.80 and conditional R^2^ values near 0.95 (Supplementary Table 4). More complex models did not improve predictive performance (Supplementary Table 5).

Accordingly, the parsimonious model was selected as the final model; the coefficients and model performance characteristics are presented in Table 2. Higher index proteinuria was significantly associated with a faster rate of eGFR decline. For each 1 g/day increase in proteinuria above the population mean, the annual rate of eGFR decline was greater by 1.47 mL/min/1.73 m^2^/year (95% confidence interval -2.76, -0.18, p = 0.009). The interaction between index eGFR and time was not statistically significant, indicating that baseline eGFR did not independently predict the rate of subsequent decline after accounting for proteinuria. Apparent model fit was excellent with marginal R^2^ was 0.81, conditional R^2^ was 0.95, and RMSE 7.25.

**Table 2.** Parameter Estimates for Parsimonious Mixed-Effects Linear Regression Model for eGFR

Predictor	Estimated coefficient	Standard error	95% CI	p-value
Marginal Intercept	**54.67**	**1.71**	**51.32, 58.02**	**<0.001**
Time	**-4.08**	**0.90**	**-5.84, -2.32**	**<0.001**
Index eGFR	**0.96**	**0.019**	**0.92, 1.00**	**<0.001**
Index Proteinuria	-0.50	0.43	-1.34, 0.34	0.251
Age	**-0.096**	**0.040**	**-0.17, -0.018**	**0.018**
Sex	-0.20	1.16	-2.47, 2.07	0.860
Index proteinuria*time	**-1.47**	**0.66**	**-2.76, -0.18**	**0.009**
Index eGFR*time	-0.0090	0.027	-0.062, 0.044	0.743
SD(Intercept|Patient)	6.21	​	​	​
SD(Time|Patient)	9.86	​	​	​
SD(Observations)	7.29	​	​	​
AIC	​	​	​	145534
BIC	​	​	​	145629
Marginal R^2^	​	​	​	0.81
Conditional R^2^	​	​	​	0.95
RMSE	​	​	​	7.25

Continuous covariates were mean-centred prior to model fitting; mean index eGFR was 57 mL/min/1.73 m^2^, mean index proteinuria was 1.58 g/day, and mean age was 43 years.

The marginal intercept represents the expected index eGFR for a male patient with age, index eGFR, and index proteinuria equal to the population mean. Coefficients for the main effects of index eGFR, index proteinuria, and age represent the expected change in index eGFR associated with a one-unit increase above the population mean for each variable. The coefficient for sex represents the expected difference in index eGFR for female compared with male patients.

The coefficient for the main effect of time represents the population-average annual rate of change in eGFR. Interaction terms between time and baseline covariates describe how patient-specific characteristics modify the rate of eGFR decline over time. Specifically, the coefficient for index proteinuria × time represents the change in annual eGFR slope associated with each 1 g/day increase in index proteinuria above the population mean, while the coefficient for index eGFR × time represents the change in annual eGFR slope associated with each 1 mL/min/1.73 m^2^ increase in index eGFR above the population mean.

To estimate the expected annual eGFR slope for an individual patient, the mean-centred value of each covariate is multiplied by its corresponding interaction coefficient and added to the population-average slope (time coefficient).

Random intercept and random slope terms account for between-patient variability in baseline eGFR and longitudinal eGFR trajectory, respectively. Marginal R^2^ represents the variance explained by fixed effects alone, whereas conditional R^2^ represents the variance explained by both fixed and random effects.

eGFR, estimated glomerular filtration rate; SD, standard deviation; AIC, Akaike information criterion; BIC, Bayesian information criterion; RMSE, root mean squared error; CI, confidence interval.

Values that are statistically significant at α = .05 are presented in bold.

Predicted eGFR slopes were compared with observed slopes calculated for each patient. Conditional (patient-specific) predictions demonstrated strong agreement with observed slopes. In the development dataset, 88% of predicted slopes were within 1 mL/min/1.73 m^2^/year of the observed slope. Calibration plots showed close alignment between predicted and observed values (Supplementary Figure 2), and Bland-Altman analysis demonstrated good overall agreement between predicted and observed slopes, with differences centred on zero, and few predictions falling outside the 95% limits of agreement (Supplementary Figure 3).

In contrast, marginal (population-average) predictions performed poorly. Only 11% of marginally predicted slopes were within 1 mL/min/1.73 m^2^/year of observed values. Calibration was suboptimal, with a calibration slope of 1.58 (95% confidence interval 0.71–2.44), and predictions often deviated markedly from observed values across all observed values of eGFR (Supplementary Figure 4). Bland-Altman plots comparing predicted and observed eGFR slope demonstrated a negative proportional bias (Supplementary Figure 5), suggesting the marginal parsimonious model underestimated eGFR slope for patients with the most negative eGFR slope (i.e it predicted a less negative slope), and overestimates eGFR slope for patients with observed slopes close to or greater than 0 (i.e it predicted decline when it didn’t occur). The limits of agreement were also much wider than the plot of conditional predictions, indicating greater prediction error.

The apparent and cross-validated performance metrics of the parsimonious model are presented in Table 3. For the conditional eGFR slope predictions, the pooled cross-validated RMSE was 12.11 (standard deviation, SD 5.40), with an optimism estimate of 5.70. The cross-validated correlation between observed and predicted eGFR slope was -0.15 (SD 0.24), with optimism of 1.03. The cross-validated calibration slope was 0.79 (SD 21.92), with optimism of 0.35.

**Table 3.** Apparent and Cross-Validated Performance Metrics of eGFR Slope Prediction Model

Performance metric	Apparent performance	Standard error	Cross-validated performance	Standard deviation	Optimism
Conditional predictions					
RMSE	6.41	​	12.11	5.40	5.70
Pearson correlation	0.88	​	-0.15	0.24	1.03
Calibration slope	1.14	0.11	0.79	21.92	0.35
Marginal predictions					
RMSE	12.31	​	11.98	4.69	0.33
Pearson correlation	0.30	​	0.24	0.46	0.06
Calibration slope	1.58	0.44	1.89	3.88	0.31

RMSE, root mean squared error.

For the marginal eGFR slope predictions, the pooled cross-validated RMSE was 11.98 (SD 4.69), with an optimism estimate of 0.33. The cross-validated correlation between observed and predicted eGFR slope was 0.24 (SD 0.46), with optimism of 0.06. The cross-validated calibration slope was 1.89 (SD 3.88), with optimism of 0.31. In summary, internal validation demonstrated substantial performance degradation for conditional predictions, with high RMSE, near-zero correlation, and unstable calibration.

In the sensitivity analysis (Supplementary Table 6), the marginal effect of time was -4.80 mL/min/1.73 m^2^/year (SE 0.84, p <0.001), which represented a slightly larger decline than in the original analysis. The two-way interaction between proteinuria and time was similar to the original model (-1.70 mL/min/1.73 m^2^/year, SE 0.51, p = 0.001). Model performance was similar, with marginal R^2^ 0.82 and conditional R^2^ 0.93, although accuracy was lower, with an RMSE of 9.12.

## Discussion

In this population-based cohort of adults with biopsy-proven IgAN, we developed and internally validated a prediction model for 2-year eGFR slope using routinely available clinical and pathologic data. The selected model demonstrated excellent in-sample performance, with proteinuria emerging as the dominant predictor of eGFR decline. However, prediction accuracy relied heavily on patient-specific random effects, and model performance deteriorated on internal validation. These findings demonstrate that strong apparent performance in slope-based models may primarily reflect the estimation of patient-specific random effects rather than generalizable associations between baseline predictors and disease progression, as evidenced by the marked deterioration in performance of marginal predictions when random effects were set to zero.

This limitation suggests a clear direction for refinement of slope-based prediction. In the derivation step, the model to predict 2-year eGFR slope appeared to perform very well, as evidenced by model fit statistics. Crucially, the accuracy of the model was heavily dependent on patient-specific random effects, estimated from all observed eGFR measurements, including eGFR measurements after the index date. As a result, the conditional model should not be interpreted as a true baseline clinical prediction model, because future information contributed to estimation of the patient-specific slopes. We nevertheless evaluated conditional predictions because mixed-effects models with BLUP-derived random effects are the standard framework for modeling longitudinal eGFR trajectories and provide an upper bound on achievable predictive performance when patient-specific trajectory information is available.^25,34^ In clinical practice, predictions of future eGFR decline must rely only on information available at or before the time of prediction. Accordingly, the marginal model, in which patient-specific random effects are set to zero, represents the clinically applicable prediction approach we evaluated. However, marginal predictions were poorly calibrated and demonstrated substantial prediction error, suggesting that the routinely available baseline clinical and pathologic variables included in our models were insufficient to accurately predict individual eGFR trajectories. The marked deterioration in performance during internal validation further supports that the strong apparent performance of the conditional model primarily reflected estimation of patient-specific random effects rather than generalizable associations between baseline predictors and subsequent disease progression.

Although the model was not tested in a geographically different population, the findings of the internal validation suggest that the model would not perform well for new patients. One potential extension of this work would be to leverage an individual’s current and historical eGFR values the index date to estimate random effects. This approach to generate individualized conditional predictions has been used successfully in other studies to predict eGFR trajectories in diabetes^35^ and lung function decline in chronic obstructive pulmonary disease,^36^ with models demonstrating good optimism-adjusted performance in cross-validation and external validation settings. A similar approach may therefore enable development of a more accurate prediction model for eGFR slope in IgAN with the potential to generalize to new patients. Such an approach is also clinically plausible, as eGFR measurements are typically available at referral, initial consultation, biopsy, and subsequent follow-up visits, with increasing numbers of measurements expected to improve patient-specific prediction.

Although marginal predictions were less optimistic on internal validation than conditional predictions, they were poorly calibrated and imprecise. Comparison of observed and predicted eGFR slopes demonstrated systematic error in marginal predictions: eGFR decline was underestimated among patients with rapid decline and overestimated among patients with stable or improving kidney function. This bias reflects regression toward the population mean when patient-specific random effects are excluded, leaving fixed effects to explain individual variation. When included covariates are only weakly associated with subsequent eGFR change, as observed in this cohort, marginal predictions cluster tightly around the average eGFR slope. Whether these weak associations are the result of a lack of power, or the inability of currently available clinical and pathologic risk factors to capture variability in eGFR decline,^9^ warrants further study.

The results reaffirm the relationship between proteinuria and subsequent eGFR decline in IgA nephropathy.^37^ Patients with higher proteinuria at one-year post-biopsy experienced substantially faster eGFR loss after adjusting for age, sex, and index eGFR. The estimated effect size, approximately 1.5 mL/min/1.73 m^2^/year faster decline per 1 g/day higher proteinuria, emphasizes the critical importance of lowering proteinuria to prevent CKD progression and kidney failure that has been demonstrated in randomized trials.^38^ Increasing baseline,^39-43^ time-averaged,^44,45^ and time-varying proteinuria^46^ are all associated with kidney disease progression; our study adds to that literature by demonstrating that the amount of proteinuria at one-year post-biopsy is also prognostic. We did not find an association between the change in proteinuria from baseline to one year and subsequent kidney function decline. This is consistent with data from the Toronto GN Registry, which demonstrated that patients with >3 g/day of proteinuria at baseline who achieved a significant reduction in proteinuria to <1 g/day had similar outcomes to patients who had <1 g/day persistently.^2^ This supports that achieving the lowest proteinuria possible, likely at any timepoint,^47^ remains the most important treatment goal in IgAN. However, it is possible that the change in proteinuria conditional on the baseline measurement may be associated with eGFR decline; our study was not designed to detect this association.

Several era-related differences were observed. Patients diagnosed in the earliest era were younger and had higher eGFR and lower proteinuria at the index date, despite similar proteinuria at cohort entry. They also had a higher prevalence of crescents, less IFTA, and were more likely to receive glucocorticoids, which may suggest earlier or more treatment-responsive disease. Diabetes and hypertension were less common in patients diagnosed in the earliest era. Increasing prevalence of diabetes and hypertension over time may partly explain the greater IFTA, lower eGFR, and higher proteinuria observed in later eras. Variability in random slopes in the mixed-effect model also increased over time, consistent with growing heterogeneity in disease trajectories. These trends may reflect evolving biopsy practices rather than true shifts in disease biology,^37,48^ with broader biopsy indications in later eras capturing a more heterogeneous population. Changes in treatment approaches and physician practice patterns may also have contributed to era-specific differences.^37,48^

This study has several strengths. First, it used a population-based cohort with comprehensive capture of biopsy-proven IgAN, minimizing referral and selection bias. Second, linkage to high-quality administrative health data and laboratory data allowed detailed characterization of clinical variables and longitudinal follow-up.^49^ Third, we applied rigorous model evaluation methods, including calibration, agreement analyses, and internal cross-validation, providing a transparent assessment of predictive performance and generalizability.

This study has some limitations. First, the sample size was modest, constrained by missing data and the requirement for sufficient longitudinal measurements to reliably estimate eGFR slope. The cohort exclusion criteria required to estimate slope reliably may may have introduced selection bias by preferentially including patients who survived without kidney failure long enough to undergo repeated laboratory assessment. Consequently, the cohort may under-represent patients with rapidly progressive disease who reached kidney failure early and patients with milder disease who underwent less intensive monitoring. Accordingly, the resulting model should be interpreted as applying to patients with IgAN who survived without kidney failure long enough to undergo longitudinal follow-up with repeated kidney function assessment sufficient to estimate eGFR slope, rather than to all patients with newly diagnosed IgAN. In addition, eGFR slopes are estimated with greater uncertainty in patients with fewer longitudinal measurements. Although requiring at least three measurements was intended to improve stability of slope estimation, stricter requirements for more frequent eGFR testing would represent a trade-off between improved precision of observed slopes and increased selection bias toward patients undergoing more intensive follow-up. Future studies using larger and more data-dense cohorts might better evaluate this balance and assess model performance among patients with more frequent longitudinal eGFR assessment. Complete case analysis may also have introduced bias if the probability of missing data was associated with disease severity, frequency of clinical follow-up, or likelihood of progression.^50^

Second, pathologic data were incomplete in earlier eras, precluding inclusion of some Oxford classification components (M and E) and limiting assessment of histopathologic contributions to slope prediction. The addition of available histopathologic variables did not meaningfully improve model performance. Given the established prognostic importance of pathology in IgAN across multiple cohort studies^1,51^ and its contribution to improved performance of the International IgA Nephropathy Prediction Tool,^9^ this finding more likely reflects limited sample size and incomplete pathology data rather than absence of incremental prognostic value.

Finally, although apparent in-sample performance was strong, predictive accuracy deteriorated on internal validation; external validation of models in independent cohorts is needed to assess model transportability.

## Conclusions

In summary, we demonstrated that eGFR slope in IgA nephropathy can be modeled accurately in-sample using routinely available clinical variables, with proteinuria emerging as the dominant predictor of decline. However, clinically applicable baseline prediction of future eGFR slope remained poor when predictions were restricted to fixed effects available at the time of prediction and the model may therefore not generalize well to new patients. Although accurate prediction of short-term eGFR slope is clinically appealing, these findings highlight the challenges inherent in developing and validating such models using currently available predictors. Future work may explore alternative approaches to estimating patient-specific random effects and leverage data from the growing number of clinical trials in IgA nephropathy, which offer the sample size and measurement density needed to support more robust prediction and improved identification of patients at highest risk of progression.

## Supplemental Material

Supplemental Material - Challenges in Predicting Estimated Glomerular Filtration Rate Slope Among Adult Patients With IgA Nephropathy: A Population-Based Cohort Study Supplemental Material for Challenges in Predicting Estimated Glomerular Filtration Rate Slope Among Adult Patients With IgA Nephropathy: A Population-Based Cohort Study by Bryce Barr, MD, MSc, Mark Canney, MD, PhD, Navdeep Tangri, MD, PhD, Lisa M. Lix, PhD in Canadian Journal of Kidney Health and Disease

Supplemental Material - Challenges in Predicting Estimated Glomerular Filtration Rate Slope Among Adult Patients With IgA Nephropathy: A Population-Based Cohort Study Supplemental Material for Challenges in Predicting Estimated Glomerular Filtration Rate Slope Among Adult Patients With IgA Nephropathy: A Population-Based Cohort Study by Bryce Barr, MD, MSc, Mark Canney, MD, PhD, Navdeep Tangri, MD, PhD, Lisa M. Lix, PhD in Canadian Journal of Kidney Health and Disease

## Data Availability

Data are available from the Manitoba Centre for Health Policy, University of Manitoba for researchers who meet the criteria for access to confidential data. All data used in our analysis are publicly available and are held by the Government of Manitoba or contracted agencies (e.g. laboratory services). Access to this data requires approval by the Committee for Harmonized Health Impact, Privacy, and Ethics Review (CHIPER), the Government of Manitoba’s Provincial Health Research Privacy Committee (PHRPC), and Shared Health Services of Manitoba. Legal restrictions prevent the use of these data without approval from each of the individual trustees. Specific data sets used in this project include: Shared Health Diagnostic Services of Manitoba (laboratory tests); Hospital Discharge Abstracts (hospitalizations); Manitoba Health Insurance Registry (registration coverage periods, demographics); Manitoba Health Medical Services (physician claims/billings); Drug Program Information Network – DPIN (pharmacy dispensing); Manitoba GN Registry (biopsy results). All of these data are provided in de-identifiable format and linked through a remote access server maintained by the Manitoba Centre for Health Policy (https://umanitoba.ca/manitoba-centre-for-health-policy/). Email address: mchp.access@umanitoba.ca.

## References

[1] Barbour SJ Espino-Hernandez G Reich HN , et al. The MEST score provides earlier risk prediction in lgA nephropathy. Kidney Int. 2016;89(1):167-175. doi:10.1038/ki.2015.322. 26759049

[2] Reich HN Troyanov S Scholey JW Cattran DC . Remission of proteinuria improves prognosis in IgA nephropathy. J Am Soc Nephrol. 2007;18(12):3177-3183. doi:10.1681/ASN.2007050526. 17978307

[3] Ai Z Zhou Q Huang F Yang Q Yu X . Long-term renal outcomes of IgA nephropathy presenting with different levels of proteinuria. Clin Nephrol. 2020;94(6):290-296. doi:10.5414/CN110192. 32990213

[4] McGrogan A Franssen CFM de Vries CS . The incidence of primary glomerulonephritis worldwide: a systematic review of the literature. Nephrol Dial Transplant Off Publ Eur Dial Transpl Assoc - Eur Ren Assoc. 2011;26(2):414-430. doi:10.1093/ndt/gfq665. 21068142

[5] Barr B Harasemiw O Gibson IW Tremblay-Savard O Tangri N . The Development of a Comprehensive Clinicopathologic Registry for Glomerular Diseases Using Natural Language Processing. Can J Kidney Heal Dis. 2023;10:20543581231178964. doi:10.1177/20543581231178963. PMC10278432 37342151

[6] Rauen T Eitner F Fitzner C , et al. Intensive Supportive Care plus Immunosuppression in IgA Nephropathy. N Engl J Med. 2015;373(23):2225-2236. doi:10.1056/NEJMoa1415463. 26630142

[7] Pozzi C Andrulli S Pani A , et al. IgA nephropathy with severe chronic renal failure: a randomized controlled trial of corticosteroids and azathioprine. J Nephrol. 2013;26(1):86-93. doi:10.5301/jn.5000110. 22460183

[8] Leaf DE Appel GB Radhakrishnan J . Glomerular disease: Why is there a dearth of high quality clinical trials. Kidney Int. 2010;78(4):337-342. doi:10.1038/ki.2010.156. 20520593

[9] Barbour SJ Coppo R Zhang H , et al. Evaluating a New International Risk-Prediction Tool in IgA Nephropathy. JAMA Intern Med. 2019;179(7):942-952. doi:10.1001/jamainternmed.2019.0600. PMC6583088 30980653

[10] Tangri N Stevens LA Griffith J , et al. A predictive model for progression of chronic kidney disease to kidney failure. JAMA. 2011;305(15):1553-1559. doi:10.1001/jama.2011.451. 21482743

[11] Pitcher D Braddon F Hendry B , et al. Long-Term Outcomes in IgA Nephropathy. Clin J Am Soc Nephrol. 2023;18:727-738. doi:10.2215/CJN.0000000000000135. PMC10278810 37055195

[12] Sim JJ Chen Q Cannizzaro N , et al. CKD progression, kidney failure, and mortality among US patients with IgA nephropathy. Nephrol Dial Transplant Off Publ Eur Dial Transpl Assoc - Eur Ren Assoc. 2025;40:2104-2117. doi:10.1093/ndt/gfaf084. PMC12559788 40307873

[13] Thompson A Smith K Lawrence J . Change in Estimated GFR and Albuminuria as End Points in Clinical Trials: A Viewpoint From the FDA. Am J kidney Dis Off J Natl Kidney Found. 2020;75(1):4-5. doi:10.1053/j.ajkd.2019.08.007. 31672253

[14] Perkovic V Barratt J Rovin B , et al. Alternative Complement Pathway Inhibition with Iptacopan in IgA Nephropathy. N Engl J Med. 2025;392(6):531-543. doi:10.1056/NEJMoa2410316. 39453772

[15] Lafayette R Kristensen J Stone A , et al. Efficacy and safety of a targeted-release formulation of budesonide in patients with primary IgA nephropathy (NefIgArd): 2-year results from a randomised phase 3 trial. Lancet (London, England). 2023;402(10405):859-870. doi:10.1016/S0140-6736(23)01554-4. 37591292

[16] Rovin BH Barratt J Heerspink HJL , et al. Efficacy and safety of sparsentan versus irbesartan in patients with IgA nephropathy (PROTECT): 2-year results from a randomised, active-controlled, phase 3 trial. Lancet. 2023;402:2077-2090. doi:10.1016/S0140-6736(23)02302-4. 37931634

[17] Komenda P Yu N Leung S , et al. Determination of the optimal case definition for the diagnosis of end-stage renal disease from administrative claims data in Manitoba, Canada. C open. 2015;3(3):E264-E269. doi:10.9778/cmajo.20140006. PMC4596097 26457290

[18] Inker LA Eneanya ND Coresh J , et al. New Creatinine- and Cystatin C-Based Equations to Estimate GFR without Race. N Engl J Med. 2021;385(19):1737-1749. doi:10.1056/NEJMoa2102953. PMC8822996 34554658

[19] Hogan MC Reich HN Nelson PJ , et al. The relatively poor correlation between random and 24-hour urine protein excretion in patients with biopsy-proven glomerular diseases. Kidney Int. 2016;90(5):1080-1089. doi:10.1016/j.kint.2016.06.020. PMC5065749 27528553

[20] Ohisa N Yoshida K Matsuki R , et al. A comparison of urinary albumin-total protein ratio to phase-contrast microscopic examination of urine sediment for differentiating glomerular and nonglomerular bleeding. Am J kidney Dis Off J Natl Kidney Found. 2008;52(2):235-241. doi:10.1053/j.ajkd.2008.04.014. 18572288

[21] Smith ER Cai MMX McMahon LP Wright DA Holt SG . The value of simultaneous measurements of urinary albumin and total protein in proteinuric patients. Nephrol Dial Transplant Off Publ Eur Dial Transpl Assoc - Eur Ren Assoc. 2012;27(4):1534-1541. doi:10.1093/ndt/gfr708. 22193048

[22] Lix LM Ayles J Bartholomew S , et al. The Canadian Chronic Disease Surveillance System: A model for collaborative surveillance. Int J Popul data Sci. 2018;3(3):433. doi:10.23889/ijpds.v3i3.433. PMC7299467 32935015

[23] Organization WH . Anatomical Therapeutic Chemical (ATC) Classification. https://www.who.int/tools/atc-ddd-toolkit/atc-classification (Accessed October 31, 2024).

[24] Trimarchi H Barratt J Cattran DC , et al. Oxford Classification of IgA nephropathy 2016: an update from the IgA Nephropathy Classification Working Group. Kidney Int. 2017;91(5):1014-1021. doi:10.1016/j.kint.2017.02.003. 28341274

[25] Vonesh E Tighiouart H Ying J , et al. Mixed‐effects models for slope‐based endpoints in clinical trials of chronic kidney disease. Stat Med. 2019;38:4218-4239. doi:10.1002/sim.8282. 31338848

[26] Portet S . A primer on model selection using the Akaike Information Criterion. Infect Dis Model. 2020;5:111-128. doi:10.1016/j.idm.2019.12.010. PMC6962709 31956740

[27] Schwarz G . Estimating the Dimension of a Model. Ann Stat. 1978;6(2):461-464. https://www.jstor.org/stable/2958889

[28] Nakagawa S Schielzeth H . A general and simple method for obtaining R2 from generalized linear mixed-effects models. Methods Ecol Evol. 2013;4(2):133-142. doi:10.1111/j.2041-210x.2012.00261.x.

[29] Hodson TO . Root-mean-square error (RMSE) or mean absolute error (MAE): when to use them or not. Geosci Model Dev. 2022;15(14):5481-5487. doi:10.5194/gmd-15-5481-2022.

[30] Van Calster B McLernon DJ van Smeden M Wynants L Steyerberg EW . Calibration: the Achilles heel of predictive analytics. BMC Med. 2019;17(1):230. doi:10.1186/s12916-019-1466-7. PMC6912996 31842878

[31] Bland JM Altman DG . Statistical methods for assessing agreement between two methods of clinical measurement. Lancet (London, England). 1986;1(8476):307-310. 2868172

[32] White IR Royston P Wood AM . Multiple imputation using chained equations: Issues and guidance for practice. Stat Med. 2011;30(4):377-399. doi:10.1002/sim.4067. 21225900

[33] Neuen B Tighiouart H Heerspink H , et al. Acute Treatment Effects on GFR in Randomized Clinical Trials of Kidney Disease Progression. J Am Soc Nephrol. 2021;33:ASN.2021070948. doi:10.1681/ASN.2021070948. PMC8819983 34862238

[34] Oshima M Gosho M Iwagami M , et al. Review no. 2: a beginner’s guide for calculating eGFR slope using linear mixed-effects model in R-step-by-step methods and code examples. Clin Exp Nephrol. 2026;30(4):545-558. doi:10.1007/s10157-026-02835-8. PMC13009037 41746487

[35] Gregorich M Kammer M Heinzel A , et al. Development and Validation of a Prediction Model for Future Estimated Glomerular Filtration Rate in People With Type 2 Diabetes and Chronic Kidney Disease. JAMA Netw open. 2023;6(4):e231870. doi:10.1001/jamanetworkopen.2023.1870. PMC10077108 37017968

[36] Zafari Z Sin DD Postma DS , et al. Individualized prediction of lung-function decline in chronic obstructive pulmonary disease. C Can Med Assoc J = J l’Association medicale Can. 2016;188(14):1004-1011. doi:10.1503/cmaj.151483. PMC5047815 27486205

[37] Rovin BH Barratt J Cook HT , et al. KDIGO 2025 Clinical Practice Guideline for the Management of Immunoglobulin A Nephropathy (IgAN) and Immunoglobulin A Vasculitis (IgAV). Kidney Int. 2025;108(4):S1-S71. doi:10.1016/j.kint.2025.04.004. 40975564

[38] Inker LA Heerspink HJL Tighiouart H , et al. Association of Treatment Effects on Early Change in Urine Protein and Treatment Effects on GFR Slope in IgA Nephropathy: An Individual Participant Meta-analysis. Am J Kidney Dis. 2021;78(3):340-349.e1. doi:10.1053/j.ajkd.2021.03.007. PMC8384669 33775708

[39] Wakai K Kawamura T Endoh M , et al. A scoring system to predict renal outcome in IgA nephropathy: from a nationwide prospective study. Nephrol Dial Transplant Off Publ Eur Dial Transpl Assoc - Eur Ren Assoc. 2006;21(10):2800-2808. doi:10.1093/ndt/gfl342. 16822793

[40] Radford MGJ Donadio JVJ Bergstralh EJ Grande JP . Predicting renal outcome in IgA nephropathy. J Am Soc Nephrol. 1997;8(2):199-207. doi:10.1681/ASN.V82199. 9048338

[41] Frimat L Briançon S Hestin D , et al. IgA nephropathy: prognostic classification of end-stage renal failure. L’Association des Néphrologues de l’Est. Nephrol Dial Transplant Off Publ Eur Dial Transpl Assoc - Eur Ren Assoc. 1997;12(12):2569-2575. doi:10.1093/ndt/12.12.2569. 9430853

[42] Lv J Zhang H Zhou Y Li G Zou W Wang H . Natural history of immunoglobulin A nephropathy and predictive factors of prognosis: a long-term follow up of 204 cases in China. Nephrology (Carlton). 2008;13(3):242-246. doi:10.1111/j.1440-1797.2007.00898.x. 18221258

[43] Bartosik LP Lajoie G Sugar L Cattran DC . Predicting progression in IgA nephropathy. Am J kidney Dis Off J Natl Kidney Found. 2001;38(4):728-735. doi:10.1053/ajkd.2001.27689. 11576875

[44] Le W Liang S Hu Y , et al. Long-term renal survival and related risk factors in patients with IgA nephropathy: results from a cohort of 1155 cases in a Chinese adult population. Nephrol Dial Transplant. 2012;27(4):1479-1485. doi:10.1093/ndt/gfr527. 21965586

[45] Berthoux F Mohey H Laurent B Mariat C Afiani A Thibaudin L . Predicting the risk for dialysis or death in IgA nephropathy. J Am Soc Nephrol. 2011;22(4):752-761. doi:10.1681/ASN.2010040355. PMC3065230 21258035

[46] Barbour SJ Cattran DC Espino-Hernandez G Hladunewich MA Reich HN . Identifying the ideal metric of proteinuria as a predictor of renal outcome in idiopathic glomerulonephritis. Kidney Int. 2015;88(6):1392-1401. doi:10.1038/ki.2015.241. 26287314

[47] Canney M Barbour SJ Zheng Y , et al. Quantifying Duration of Proteinuria Remission and Association with Clinical Outcome in IgA Nephropathy. J Am Soc Nephrol. 2021;32(2):436-447. doi:10.1681/ASN.2020030349. PMC8054888 33514642

[48] KDIGO. KDIGO 2012 . Clinical Practice Guideline for the Evaluation and Management of Chronic Kidney Disease. Kidney Int Suppl. 2013;3(1):4. doi:10.1038/kisup.2012.76. 23989362

[49] Smith M Lix LM Azimaee M , et al. Assessing the quality of administrative data for research: A framework from the manitoba centre for health policy. J Am Med Informatics Assoc. 2018;25(3):224-229. doi:10.1093/jamia/ocx078. PMC7651885 29025002

[50] Little RJA Rubin DB . Statistical Analysis with Missing Data. 2nd ed. Hoboken, NJ: Wiley; 2002.

[51] Coppo R D’Arrigo G Tripepi G , et al. Is there long-term value of pathology scoring in immunoglobulin A nephropathy? A validation study of the Oxford Classification for IgA Nephropathy (VALIGA) update. Nephrol Dial Transplant Off Publ Eur Dial Transpl Assoc - Eur Ren Assoc. 2020;35(6):1002-1009. doi:10.1093/ndt/gfy302. 30418652

