# Supplementary material for: Challenges in Predicting Estimated Glomerular Filtration Rate Slope Among Adult Patients With IgA Nephropathy: A Population-Based Cohort Study
Source: Can J Kidney Health Dis. 2026 Sep 30;13:20543581261468724. doi: 10.1177/20543581261468724 (PMC13769825; doi:10.1177/20543581261468724)
Supplement: Supplementary file 1 [file sj-pdf-1-cjk-10.1177_20543581261468724.pdf]

Supplementary Table 1. Study definitions of nephrology visit, dialysis, kidney transplantation, and comorbidities

Variable	Medical Health Services Claims Diagnosis and Tariff Codes	Hospital Discharge Abstracts Diagnosis Codes
**Nephrology Visit**	The first tariff code from the following list:<br>**Inpatient consultation**: 8595<br>**Outpatient consultation:** 8550<br>**Complete history and physical examination:** 8540<br>arising from bloc 016 (Adult Nephrology)	--
**Dialysis**	2 or more codes from the following list:<br>**Initial dialysis:** 9798, 9801, 9805, 9806<br>**Subsequent dialysis:** 9799, 9802, 9807, 9814, 9819, 9820, 9821, 9610	--
**Transplantation**	1 tariff code or diagnosis code	
	**Kidney transplant:** 5883	ICD-9-CM: 556<br>ICD-10-CA: 1PC85, 1OK85, 556
**Hypertension**	1 hospital diagnosis or 2 outpatient claims within 2 years	
		ICD-9-CM: 401, 402, 403, 404, 405<br>ICD-10-CA: I10, I11, I12, I13, I15
**Diabetes**	1 hospital diagnosis or 2 outpatient claims within 2 years	
		ICD-9-CM: 250<br>ICD-10-CA: E10, E11, E13, E14

ICD-9-CM, International Classification of Diseases, Ninth Revision, Clinical Modification

ICD-10-CA, International Classification of Diseases, Tenth Revision, Canadian Version

Supplementary Table 2. Summary of mixed-effect models for eGFR slope and their covariates

**Model**	**Covariates**	**Covariates included as interaction terms with time**
Parsimonious model	Index eGFR<br>Index Proteinuria<br>Age<br>Sex	Index eGFR<br>Index Proteinuria
Treatment and trajectory model	Model A and<br>Delta-proteinuria<br>Delta-eGFR<br>Time to biopsy<br>Treatment with RAASi<br>Treatment with steroids	Index eGFR<br>Index Proteinuria<br>Treatment with RAASi<br>Treatment with steroids
Full clinical model	Model B and<br>Hypertension<br>Diabetes	Index eGFR<br>Index Proteinuria<br>Treatment with RAASi<br>Treatment with steroids<br>Delta-eGFR
Full clinicopathologic model	Model C and<br>Oxford S<br>Oxford T<br>Oxford C	Index eGFR<br>Index Proteinuria<br>Treatment with RAASi<br>Treatment with steroids<br>Oxford S<br>Oxford T<br>Oxford C

eGFR, estimated glomerular filtration rate; RAASi, renin-angiotensin-aldosterone system inhibitor

Supplementary Table 3. Baseline characteristics of patients with IgA nephropathy by number of eGFR measurements per patient

Characteristic	2 Measurements (n = 60)	≥3 Measurements (n = 181)	Complete Cases (n = 137)
**Age at cohort entry (years)**	44 (34, 52)	41 (31, 54)	41 (30, 54)
**Sex**			
**Male**	37 (62%)	101 (56%)	81 (59%)
**Female**	23 (38%)	80 (44%)	56 (41%)
**Location of Residence**			
**Winnipeg**	41 (68%)	108 (60%)	80 (58%)
**Outside of Winnipeg**	19 (32%)	73 (40%)	57 (41%)
**SES Quintile**			
**1 (lowest)**	26 (43%)	62 (34%)	46 (34%)
**2**	11 (18%)	36 (20%)	29 (21%)
**3**	7 (12%)	30 (17%)	22 (16%)
**4**	8 (13%)	36 (20%)	28 (20%)
**5 (highest)**	8 (13%)	17 (9%)	12 (9%)
**eGFR at cohort entry (mL/min/1.73 m^2)**	48 (32, 71)	58 (35, 86)	59 (36, 88)
Missing	7 (12%)	13 (7.2%)	N/A
**Proteinuria at cohort entry (g/day)**	1.70 (0.88, 2.71)	1.73 (1.06, 2.95)	1.77 (1.09, 2.96)
Missing	9 (15%)	14 (7.7 %)	N/A
**Index eGFR (mL/min/1.73 m^2)**	40 (20, 82)	51 (31, 81)	53 (32, 82)
Missing	27 (45%)	26 (14%)	N/A
**Index proteinuria (g/day)**	0.83 (0.37, 1.57)	1.19 (0.53, 2.27)	1.15 (0.55, 2.28)
Missing	16 (27%)	33 (18%)	N/A
**Nephrology consult to biopsy (days)**	51 (13, 382)	49 (8, 210)	40 (6, 171)
**Diabetes**	9 (15%)	31 (17%)	22 (16%)
**Hypertension**	31 (52%)	83 (46%)	59 (43%)
**RAASi treatment**	46 (77%)	160 (88%)	122 (89%)
**Glucocorticoid treatment**	8 (13%)	46 (25%)	41 (30%)
**Oxford S**			
**0**	26 (43%)	50 (28%)	39 (28%)
**1**	34 (57%)	131 (72%)	98 (72%)
**Oxford T**			
**0**	32 (53%)	94 (52%)	69 (50%)
**1**	9 (15%)	57 (31%)	47 (34%)
**2**	19 (32%)	30 (17%)	21 (15%)
**Oxford C (binary)**			
**0**	51 (85%)	132 (73%)	95 (69%)
**1**	9 (15%)	49 (27%)	42 (31%)
**Kidney failure status**			
**No kidney failure**	48 (80%)	148 (82%)	116 (85%)
**Kidney failure**	12 (20%)	33 (18%)	21 (15%)
**Vital status**			
**Alive**	54 (90%)	155 (86%)	119 (87%)

**Dead**	6 (10%)	26 (14%)	18 (13%)
**Follow up time (years)**	7.1 (4.0, 10.6)	8.2 (5.2, 12.6)	8.3 (5.2, 12.8)

Results are reported as median (Q1, Q3) for continuous variables, and n (%) for categorical variables
Some percentages may not sum to 100 due to rounding error.

SES, socioeconomic status; eGFR, estimated glomerular filtration rate; RAASi, renin-angiotensin-aldosterone system inhibitor; N/A, not applicable

Supplementary Table 4. Coefficients, 95% confidence intervals, and model performance measures for mixed-effects linear regression models for eGFR slope

Predictor	Parsimonious	Treatment and trajectory	Full Clinical	Full Clinicopathologic
Marginal Intercept	**54.67 (51.32, 58.02)**	**53.63 (50.00, 57.26)**	**52.90 (48.98, 56.82)**	**51.38 (46.42, 56.34)**
Time	**-4.08 (-5.84, -2.32)**	**-4.43 (-6.74, -2.12)**	**-5.29 (-8.07, -2.51)**	-1.66 (-6.38, 3.06)
Index eGFR	**0.96 (0.92, 1.00)**	**0.96 (0.92, 1.00)**	**0.96 (0.92, 1.00)**	**0.96 (0.91, 1.00)**
Index Proteinuria	-0.50 (-1.34, 0.34)	-0.73 (-1.67, 0.21)	-0.86 (-1.84, 0.12)	-0.97 (-1.95, 0.01)
Age	**-0.096 (-0.17, -0.018)**	-0.078 (-0.16, 0.01)	-0.086 (-0.18, 0.004)	-0.079 (-0.17, 0.011)
Sex	-0.20 (-2.47, 2.07)	-0.32 (-2.73, 2.09)	-0.35 (-2.78, 2.08)	-0.39 (-2.86, 2.08)
Delta-proteinuria		0.092 (-0.38, 0.56)	0.10 (-0.37, 0.57)	0.044 (-0.43, 0.51)
Delta-eGFR		0.057 (-0.027, 0.14)	0.076 (-0.014, 0.17)	0.076 (-0.018, 0.17)
Duration of time to biopsy (days)		**0.0016 (-0.00013, 0.0031)**	**0.0018 (0.00029, 0.0033)**	**0.0019 (0.00035, 0.0034)**
Corticosteroids		1.73 (-0.88, 4.34)	1.60 (-1.05, 4.25)	1.48 (-1.34, 4.30)
Hypertension			1.38 (-1.21, 3.97)	1.54 (-1.07, 4.15)
Diabetes			-0.70 (-4.09, 2.69)	-0.85 (-4.46, 2.76)
Oxford S				2.59 (-0.15, 5.33)
Oxford T1				-0.37 (-3.35, 2.61)
Oxford T2				-0.69 (-4.47, 3.09)
Oxford C1 or 2				-0.37 (-3.39, 2.65)
Index proteinuria*time	**-1.47 (-2.76, -0.18)**	**-1.72 (-3.13, -0.31)**	**-1.92 (-3.37, -0.47)**	**-1.78 (-3.23, -0.33)**
Index eGFR*time	-0.0090 (-0.062, 0.044)	-0.011 (-0.070, 0.048)	-0.013 (-0.072, 0.046)	-0.028 (-0.093, 0.037)
Steroids*time		0.86 (-3.14, 4.86)	0.12 (-4.09, 4.33)	1.84 (-2.57, 6.25)
Delta-eGFR*time			-0.073 (-0.20, 0.059)	0.087 (-0.048, 0.22)
Oxford S*time				-2.35 (-6.60, 1.90)
Oxford T1*time				-1.34 (-5.77, 3.09)
Oxford T2*time				-4.38 (-10.22, 1.46)
Oxford C*time				**-4.61 (-9.16, -0.063)**
SD(Intercept|Patient)	6.21	6.08	6.31	6.31
SD(Time|Patient)	9.86	11.236	10.24	10.05
SD(Observations)	7.29	7.31	7.35	7.35
AIC	145534	145082	145084	145063
BIC	145629	145217	145243	145286
Marginal R^2	0.81	0.81	0.80	0.80
Conditional R^2	0.95	0.95	0.95	0.95
RMSE	7.25	7.31	7.31	7.31

eGFR, estimated glomerular filtration rate; SD, standard deviation; AIC, Akaike information criterion; BIC, Bayesian information criterion; RMSE, root mean squared error. Values that are statistically significant at $\alpha = .05$ are presented in boldface font

Supplementary Table 5. Apparent performance metrics for eGFR slope predictions generated from mixed-effect linear regression models

Metric	Parsimonious model	Treatment and trajectory model	Full clinical model	Full clinicopathologic model
Conditional				
RMSE	6.41	6.54	6.31	6.93
Correlation	0.88	0.88	0.88	0.87
Calibration slope (95% CI)	1.15 (1.04, 1.26)	1.14 (1.03, 1.26)	1.14 (1.04, 1.25)	1.14 (1.02, 1.26)
Within 1 mL/min/year	88%	83%	69%	74%
Marginal				
RMSE	12.31	12.69	12.65	12.60
Correlation	0.30	0.28	0.28	0.31
Calibration slope (95% CI)	1.58 (0.71, 2.44)	1.55 (0.60, 2.51)	1.39 (0.54, 2.25)	1.19 (0.54, 1.84)
Within 1 mL/min/year	11%	10%	14%	10%

RMSE, root mean squared error; CI, confidence interval

Supplementary Table 6. Coefficients and standard errors from parsimonious mixed-effects linear regression model for eGFR fit with index date set at 180 days post-kidney biopsy

Predictor	Coefficient	Standard error	95% CI	p-value
Marginal Intercept	**56.98**	**1.66**	**53.73, 60.23**	**<0.001**
Time	**-4.80**	**0.84**	**-6.45, -3.15**	**<0.001**
Index eGFR	**0.97**	**0.019**	**0.93, 1.00**	**<0.001**
Index Proteinuria	**-1.01**	**0.34**	**-1.68, -0.34**	**0.004**
Age	**0.057**	0.039	-0.019, 0.13	0.147
Sex	0.29	1.12	-1.91, 2.49	0.798
Index proteinuria*time	**-1.70**	**0.51**	**-2.70, -0.70**	**0.001**
Index eGFR*time	-0.012	0.025	-0.061, 0.037	0.637
SD(Intercept|Patient)	6.34			
SD(Time|Patient)	10.01			
SD(Observations)	9.16			
AIC				205910
BIC				206009
Marginal R^2				0.82
Conditional R^2				0.93
RMSE				9.12

eGFR, estimated glomerular filtration rate; SD, standard deviation; AIC, Akaike information criterion; BIC, Bayesian information criterion; RMSE, root mean squared error. Values that are statistically significant at $\alpha = .05$ are presented in boldface font

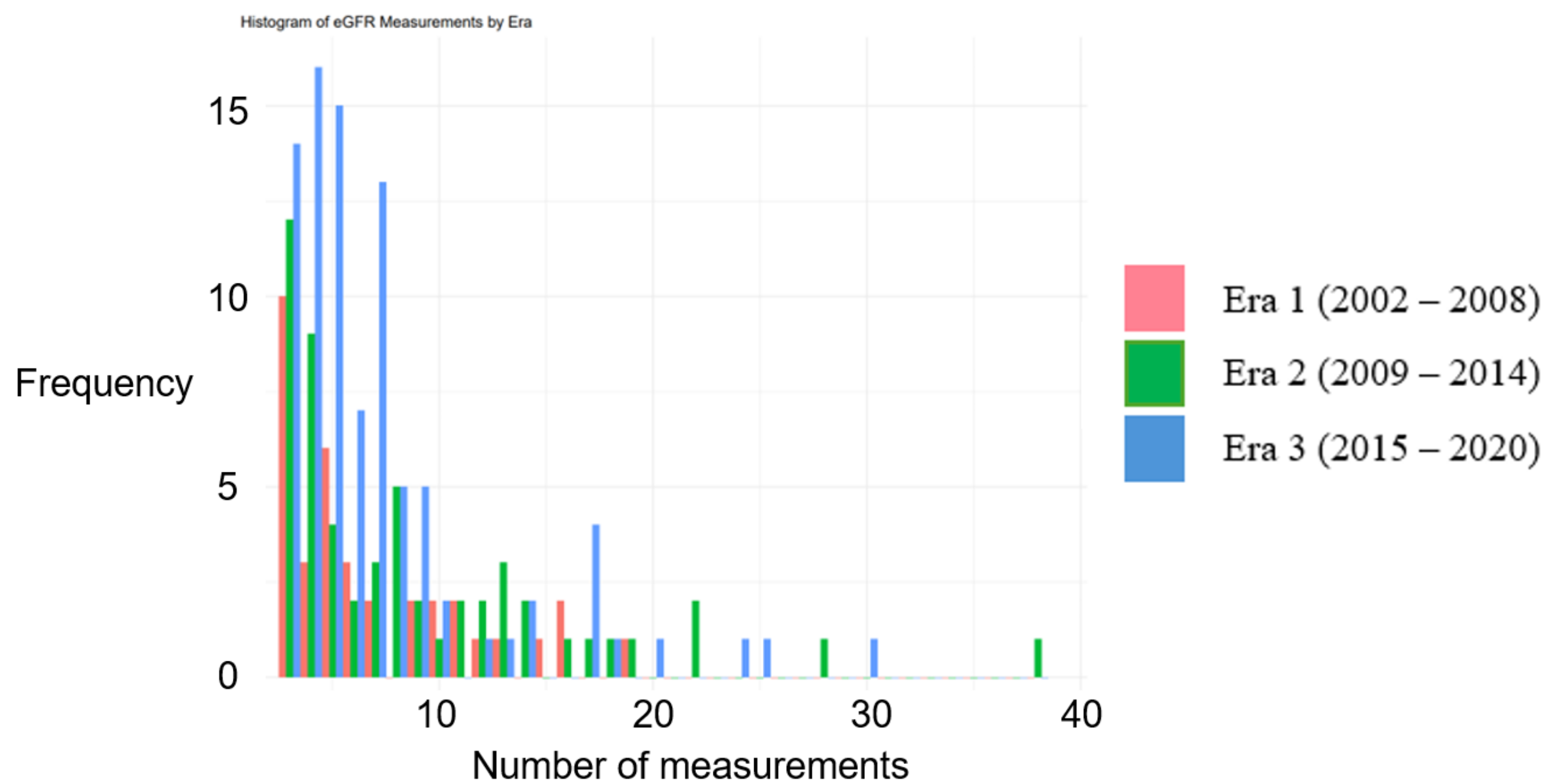

Supplementary Figure 1. Frequency of eGFR measurements per patient within two years of index date, stratified by era

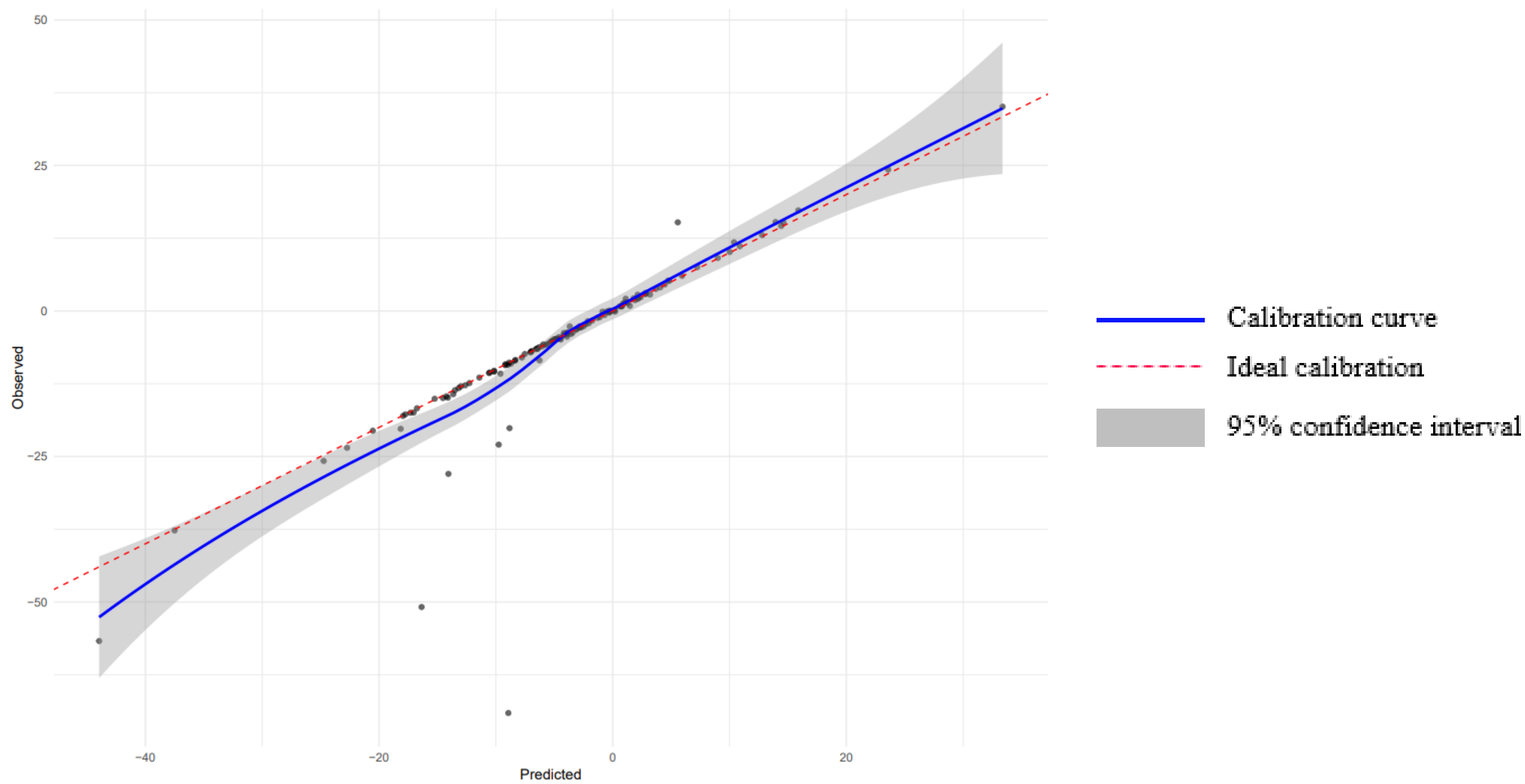

Supplementary Figure 2. Calibration plot of conditional predicted versus observed eGFR slopes from the parsimonious mixed-effects linear regression model

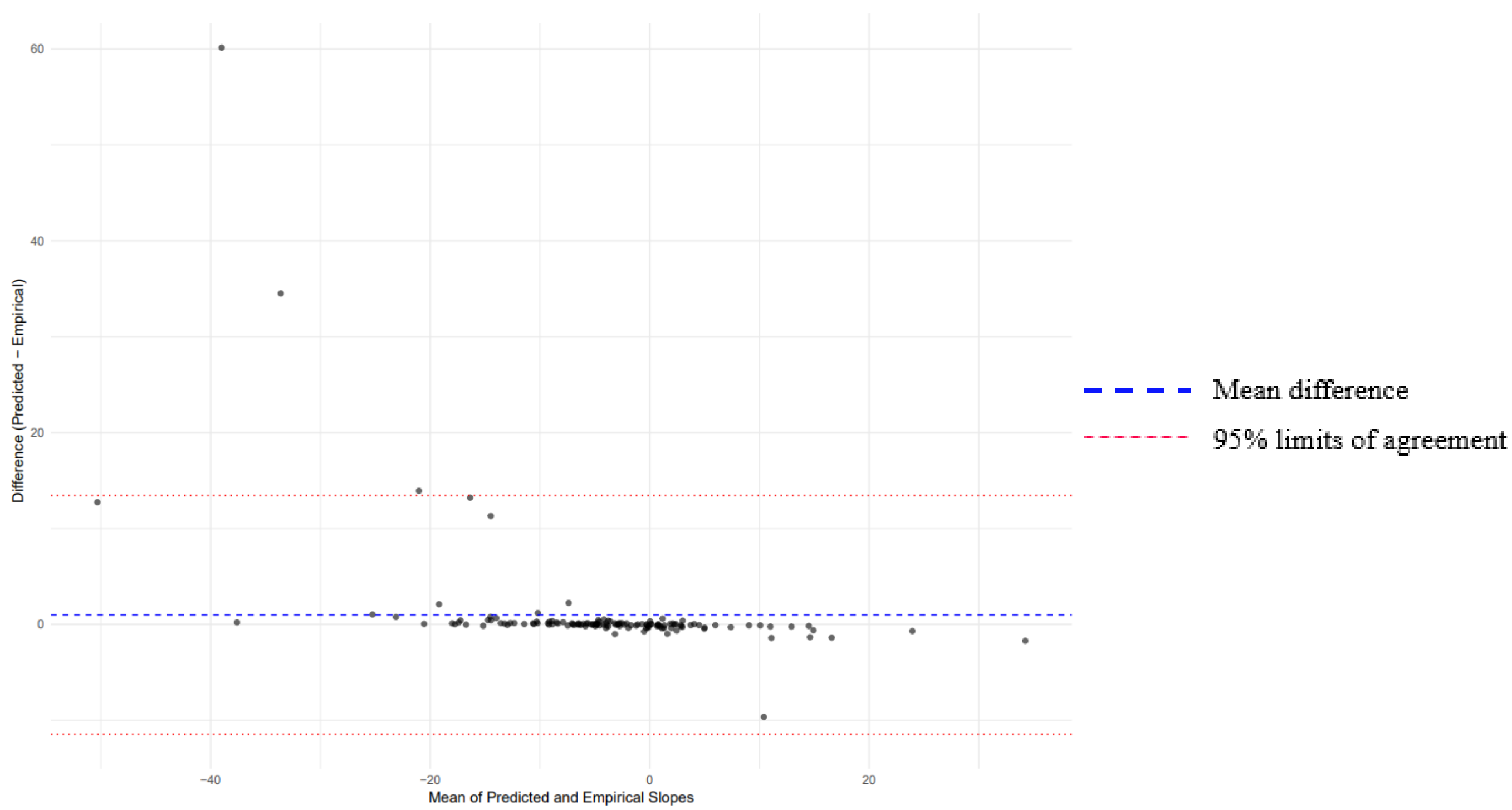

Supplementary Figure 3. Bland–Altman analysis of conditional predicted versus observed eGFR slopes from the parsimonious mixed-effects linear regression model

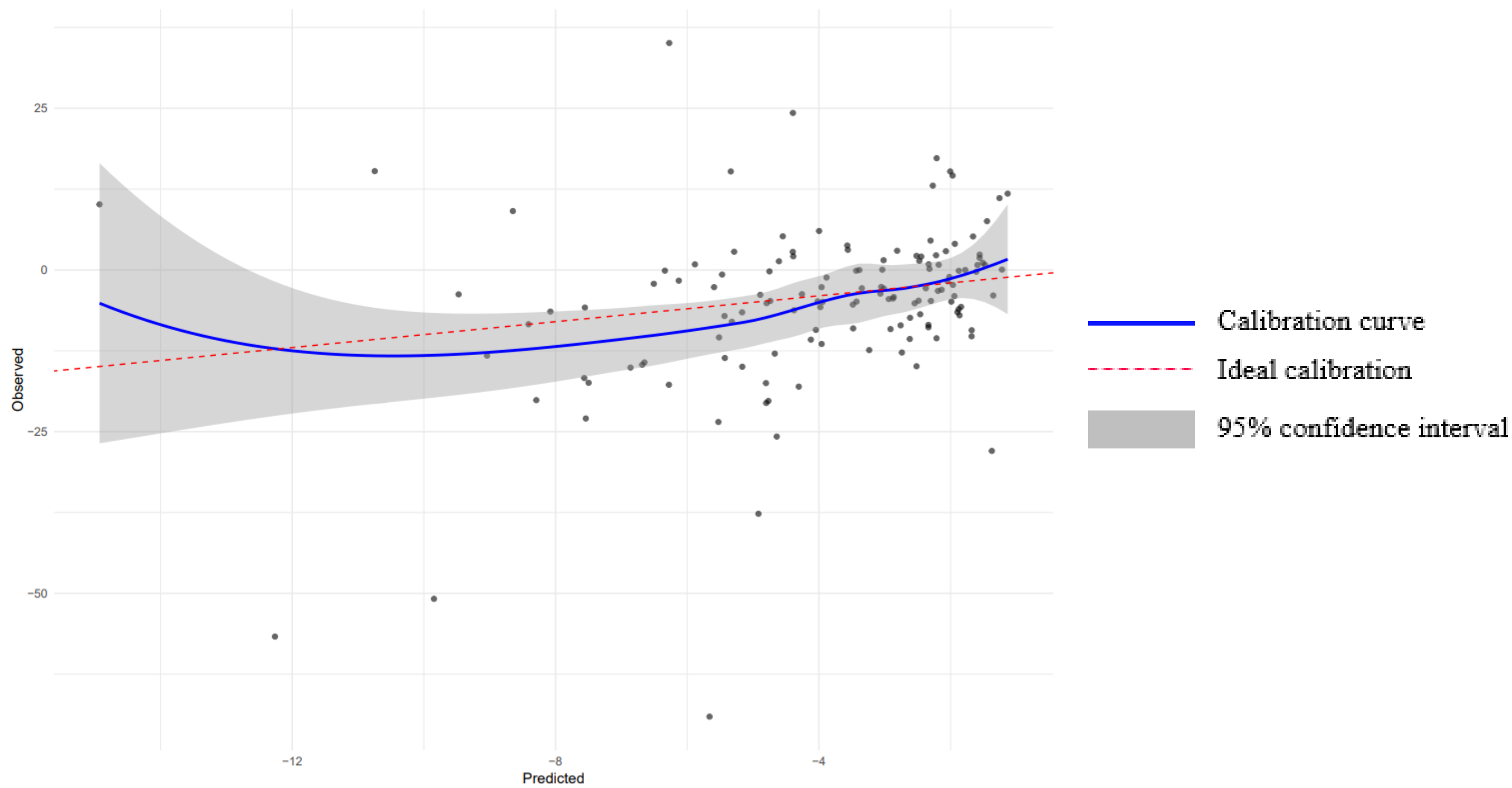

Supplementary Figure 4. Calibration plot of marginal predicted versus observed eGFR slopes from the parsimonious mixed-effects linear regression model

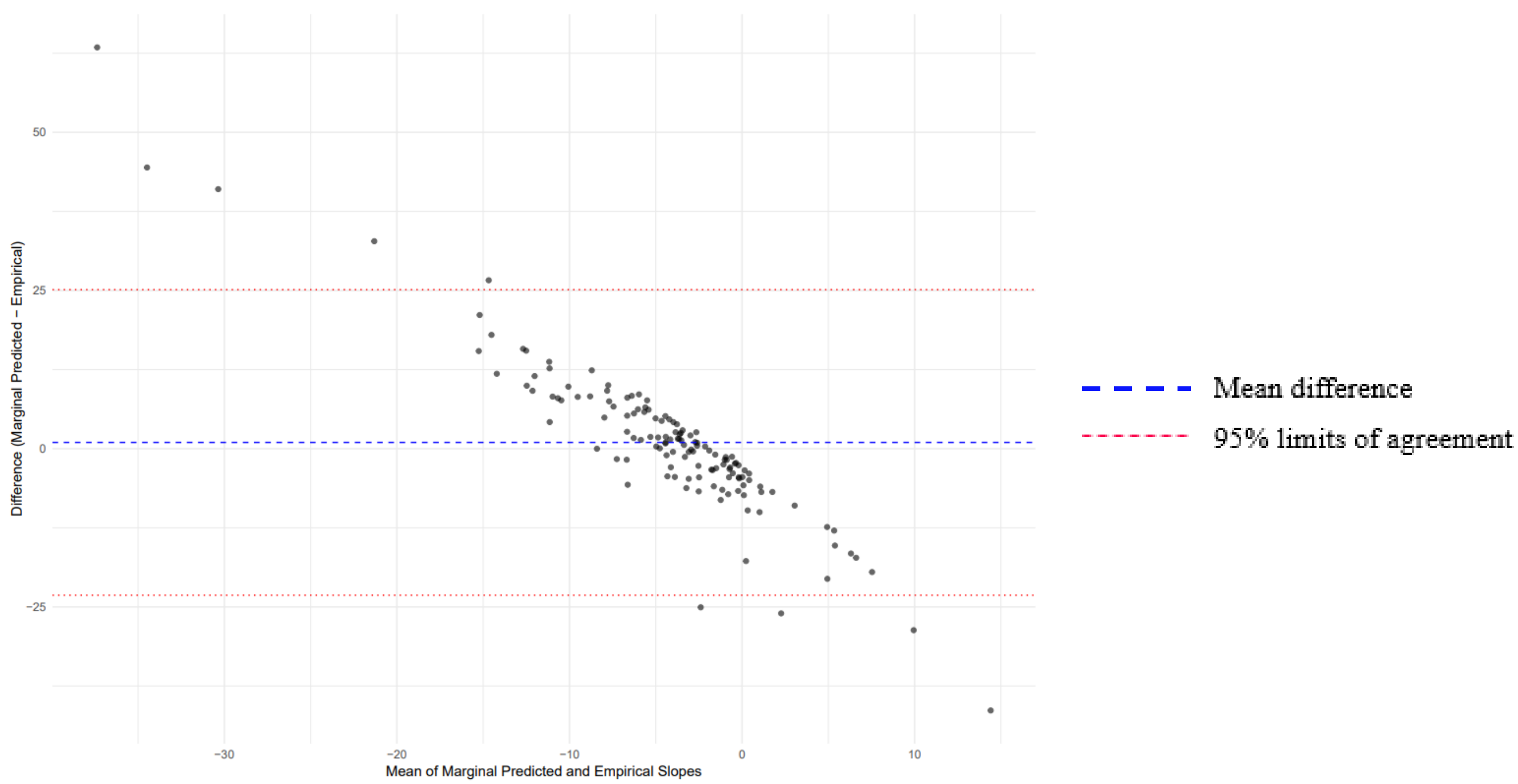

Supplementary Figure 5. Bland–Altman analysis of marginal predicted versus observed eGFR slopes from the parsimonious mixed-effects linear regression model

Supplementary Methods

Let *i* index patients and *j* index eGFR measurements. Time was measured in years since the index date, and continuous covariates were mean-centred. Mean index eGFR was 57 mL/min/1.73 m^2, mean index proteinuria was 1.58 g/day, and mean age was 43 years.

Conditional longitudinal eGFR model

$$\begin{aligned} eGFR_{ij} = 54.67 + b_{0i} \\ &+ (-4.08 + b_{1i})Time_{ij} \\ &+ 0.96(Index\ eGFR_i) \\ &- 0.50(Index\ proteinuria_i) \\ &- 0.096(Age_i) \\ &- 0.20(Sex_i) \\ &- 1.47(Index\ proteinuria_i \times Time_{ij}) \\ &- 0.0090(Index\ eGFR_i \times Time_{ij}) \\ &+ \varepsilon_{ij} \end{aligned}$$

Where numerical values are the estimated values for the fixed-effect coefficients, b_{0i} is the estimated patient-specific random intercept, b_{1i} is the estimated patient-specific random slope for time, and ε_{ij} is the residual error term

Patient-specific predicted eGFR slope

Because slope is the coefficient of time, the conditional predicted annual eGFR slope for patient *I* is:

$$\widehat{slope}_i = -4.08 - 1.47(Index\ proteinuria_i) - 0.0090(Index\ eGFR_i) + b_{1i}$$

For marginal predictions, the random slope is set to zero:

$$\widehat{slope}_{i,marginal} = -4.08 - 1.47(Index\ proteinuria_i) - 0.0090(Index\ eGFR_i)$$

Because index eGFR and index proteinuria were mean-centred, the coefficient for time represents the expected annual eGFR slope for a patient with average index eGFR and proteinuria, before adding patient-specific random effects.
