# Supplementary material for: Challenges in Predicting Estimated Glomerular Filtration Rate Slope Among Adult Patients With IgA Nephropathy: A Population-Based Cohort Study
Source: Can J Kidney Health Dis. 2026 Sep 30;13:20543581261468724. doi: 10.1177/20543581261468724 (PMC13769825; doi:10.1177/20543581261468724)
Supplement: Supplementary file 2 [file sj-pdf-2-cjk-10.1177_20543581261468724.pdf]

CJKHD-26-0020.R1

Challenges in predicting estimated glomerular filtration rate slope among adult patients with IgA nephropathy: a population-based cohort study

Résumé

Contexte : Les études sur la néphropathie à IgA (IgAN) ont traditionnellement retenu l'insuffisance rénale terminale ou le déclin du DFGe comme principaux critères de jugement. Bien que la pente du DFGe sur deux ans soit désormais reconnue comme un critère substitut validé pour la stratification précoce du risque, aucun modèle prédictif n'est actuellement disponible.

Objectifs : Élaborer et valider en interne un modèle prédictif de l'évolution du DFGe sur deux ans utilisant les données cliniques et histopathologiques couramment disponibles chez les adultes atteints d'IgAN.

Conception : Des adultes atteints d'IgAN confirmée par biopsie (2002–2021) ont été répertoriés dans le Registre manitobain des maladies glomérulaires et appariés à des données populationnelles de laboratoire et de santé administratives. Des modèles de régression linéaire à effets mixtes, intégrant des covariables démographiques, cliniques et de comorbidités ainsi que des intercepts et des pentes aléatoires, ont été utilisés pour prédire l'évolution de la pente du DFGe. Quatre modèles préspécifiés de complexité croissante ont été testés. La capacité prédictive du modèle a été évaluée à l'aide des coefficients R^2 marginal et conditionnel, de la racine de l'erreur quadratique moyenne, ainsi que d'analyses de calibration et de Bland–Altmann. La validation interne a été effectuée en utilisant une validation croisée de 10 fois.

Résultats : La cohorte réunissait 181 patients (âge médian : 41 ans; intervalle interquartile [IIQ] : 31 à 54 ans]) desquels 137 (75 %) avec des données complètes ont

été retenus pour l'analyse primaire. Lors de la biopsie, les patients présentaient un risque élevé, avec des valeurs médianes de DFGe à 58 ml/min/1,73 m^2 (IIQ : 35–86) et de protéinurie à 1,73 g/jour (IIQ : 1,06–2,95). Une protéinurie initiale élevée était indépendamment associée à un déclin plus rapide du DFGe (−1,47 ml/min/1,73 m^2/an pour chaque augmentation de 1 g/jour de protéinurie; erreur standard [ES] : 0,66; p = 0,009). Le modèle plus parcimonieux, intégrant le temps, l'âge, le sexe, le DFGe initial, la protéinurie initiale et les interactions temps–DFGe et temps–protéinurie, a montré une excellente adéquation aux données (R^2 conditionnel de 0,95). Les estimations conditionnelles de la pente (propres à chaque patient), qui intégraient des effets aléatoires individuels estimés à partir des mesures longitudinales du DFGe, reproduisaient fidèlement les trajectoires observées; 88 % des pentes prédites étant à moins de 1 ml/min/1,73 m^2/an des valeurs observées. En revanche, les prédictions marginales (moyennes populationnelles) ont montré une calibration insuffisante; seules 11 % des prédictions se situaient dans cette même marge d'erreur (pente de calibration : 1,58; IC à 95 % : 0,71–2,44). La validation croisée a montré une forte dégradation de la performance des modèles conditionnels, tandis que les prédictions marginales restaient stables, mais d'une précision insuffisante.

Limites : Les performances du modèle étaient supérieures lorsque celui-ci intégrait les données de suivi du DFGe après la date d'inclusion, ce qui en limite l'utilisation chez les patients dont le diagnostic est récent. La taille de l'échantillon était limitée par des données manquantes, et les scores Oxford M et E n'étaient pas disponibles pour tous les patients.

Conclusion : Les modèles à effets mixtes reproduisent fidèlement les trajectoires longitudinales du DFGe, mais leurs performances prédictives restent largement tributaires d'effets aléatoires individuels, ce qui limite leur généralisation aux patients nouvellement évalués. À elles seules, les caractéristiques cliniques et pathologiques initiales n'étaient pas suffisantes pour prédire avec précision la pente du DFGe. Ces résultats soulignent le potentiel et les défis des modèles prédictifs fondés sur la pente du DFGe en contexte d'IgAN. Ils mettent également en lumière le besoin de cohortes plus importantes, de mesures répétées du DFGe et de modèles intégrant les données antérieures des patients pour améliorer les performances prédictives.

Mots clés : Néphropathie à IgA; prédiction; épidémiologie; pente du DFGe; glomérulonéphrite
